# Characterizing Canine Frontal Electroencephalographic Patterns and Cardiovascular Correlates at Different Anesthetic Levels of Sevoflurane

**DOI:** 10.3390/ani15050715

**Published:** 2025-03-02

**Authors:** Carla Murillo, Jeff C. Ko, Ann B. Weil, Matthias Kreuzer, George E. Moore

**Affiliations:** 1 Department of Veterinary Clinical Sciences, College of Veterinary Medicine, Purdue University, West Lafayette, IN 47907, USA; murilloc@purdue.edu (C.M.); aweil@purdue.edu (A.B.W.); 2 Department of Anesthesiology and Intensive Care, School of Medicine and Health, Technical University of Munich, 80333 Munich, Germany; m.kreuzer@tum.de; 3 Department of Veterinary Administration, College of Veterinary Medicine, Purdue University, West Lafayette, IN 47907, USA; gemoore@purdue.edu

**Keywords:** sevoflurane, electroencephalography, EEG patterns, antinociception, minimum alveolar concentration, patient state index, burst suppression ratio, anesthetic depth, monitoring, spectral edge frequency

## Abstract

This study evaluated electroencephalogram [EEG] and cardiovascular correlation of sevoflurane anesthesia in healthy beagle dogs at varying minimum alveolar concentration (MAC) multiples. Processed EEG indices (Patient State Index [PSI], burst suppression ratio [SR], and Spectral Edge Frequency [SEF95], cardiovascular parameters (mean blood pressure [MBP], heart rate [HR]), and responses to noxious (electrical) stimuli were recorded. Deep anesthesia (2.5x MAC) resulted in the lowest PSI and MBP values (13.5 ± 9.9, 42.2 ± 7.4 mmHg, respectively), the highest SR (52.7 ± 35.4%), and dominant burst suppression. Surgical anesthesia (1.5x MAC) was characterized by alpha/low beta waves and the absence of response to noxious stimuli. At 1x MAC (2.1%), PSI and MBP increased (41.9 ± 12.6, 119.9 ± 17.7, respectively) while SR decreased (7.1 ± 13%). A moderate PSI-MBP correlation (ρ = 0.48, *p* = 0.002) was observed. Recovery was acceptable or smooth in most dogs. These findings suggest that sevoflurane disrupts cortical communication, inducing anesthesia and antinociception and that real-time EEG monitoring may aid in titrating sevoflurane delivery in conjunction with MBP.

## Simple Summary

This study investigated brain activity (using frontal electroencephalogram [EEG]) and cardiovascular responses in dogs anesthetized with different concentrations of sevoflurane. We found that EEG patterns changed predictably with increasing anesthetic levels, progressing from awake patterns to burst suppression and electrical silence. These EEG changes correlated with blood pressure but not heart rate, suggesting blood pressure may be a better indicator of anesthetic level in dogs. Our findings also reveal distinct brainwave features associated with sevoflurane anesthesia, suggesting this drug disrupts cortical communication to induce anesthesia and antinociception. This highlights the potential of real-time EEG monitoring with blood pressure to individualize anesthesia and improve procedural outcomes in dogs.

## 1. Introduction

Sevoflurane is a widely used inhalational anesthetic in human medicine. Although it has been available in the US veterinary market since 1999 [1], it is not widely accepted as in human anesthesia. Sevoflurane has several favorable pharmacokinetic properties and clinical advantages over isoflurane. Sevoflurane produces rapid induction and recovery times, making it a suitable choice for surgical procedures of various durations [1,2,3,4]. In addition, its low blood–gas partition coefficient facilitates intraoperative rapid change in the anesthetic level with greater control and easy titration [1,2,3,4,5]. Compared to isoflurane, sevoflurane offers a smoother and more rapid emergence from anesthesia, making it particularly advantageous in outpatient settings and for procedures requiring quick turnover. Additionally, its less pungent nature and reduced airway irritation make it more suitable for mask inductions, enhancing patient comfort and compliance [1,2,3,4,5].

Studies have demonstrated that sevoflurane maintains cardiovascular stability better than isoflurane, with less pronounced effects on mean arterial pressure (MBP) and heart rate (HR) at the same MAC levels [6,7,8]. This is particularly beneficial in patients with pre-existing cardiovascular conditions or those undergoing lengthy surgical procedures. Furthermore, sevoflurane has been associated with neuroprotective effects, potentially due to its ability to modulate cerebral blood flow and reduce neuronal excitotoxicity [7,8].

Electroencephalography (EEG) has emerged as a valuable tool for monitoring anesthetic level or “depth”, providing real-time insights into the brain’s response to anesthetics in both humans [9,10,11,12,13,14] and dogs [15,16,17,18,19]. Given all the significant advantages of sevoflurane in the central nervous and cardiovascular systems, its impact on EEG patterns and cardiovascular parameters at various minimum alveolar concentration (MAC) multiples deserve to be explored. We recently conducted a study evaluating the effects of isoflurane on canine EEG indices, MBP, HR, and response to nociceptive stimuli [19]. Building upon this foundation, the current study aims to explore sevoflurane’s effects using similar methodologies.

This study aimed to characterize the EEG and cardiovascular effects of sevoflurane anesthesia in dogs across different anesthetic levels. We sought to (1) describe how frontal EEG patterns and indices, including the (Patient State Index [PSI], burst suppression ratio [SR], and Spectral Edge Frequency [SEF95], change with varying sevoflurane concentrations (expressed as minimum alveolar concentration multiples); and (2) determine the correlation between PSI and cardiovascular parameters (MBP and HR). We hypothesized that frontal EEG patterns would accurately track changes in brain state associated with different MAC multiples of sevoflurane and that PSI would correlate with MBP and HR.

## 2. Materials and Methods

### 2.1. Animals

In this prospective study, six healthy male beagle dogs, each 16.5 months old and weighing between 10.5 and 13.5 kg, were used. For inclusion in the study, all dogs underwent a thorough physical examination and various tests, including a complete blood cell count, serum biochemistry, and fecal and urine analyses. The night before the experiment, the dogs were fasted for eight hours but had free access to water. The study protocol and animal use procedures were approved by the Purdue University Animal Care and Use Committee (protocol # 2108002178).

### 2.2. Experimental Design and Treatment Timeline

**Figure 1:**
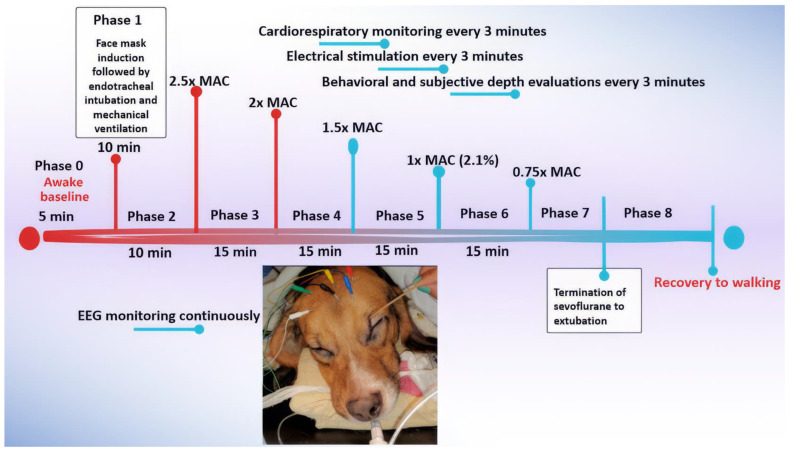
Timeline of the experimental protocol used in the current study. Six dogs were anesthetized with sevoflurane at various levels, defined by MAC multiples. The timeline shows the duration of each experimental phase, from Phase 0 (awake baseline) to Phase 8 (recovery to walking), along with key data points collected, including EEG readings, cardiorespiratory measurements, behavioral observations, and subjective anesthetic “depth” scores.

The dogs were exposed to sevoflurane starting from an awake baseline (Phase 0) and underwent face mask induction (Phase 1) with sevoflurane. The anesthetic concentration was initially set at 2.5x MAC (Phase 2) and then gradually decreased to 0.75x MAC (Phase 3–6) during the maintenance of anesthesia. Recovery was monitored until the dogs were extubated (Phase 7) and able to achieve sternal recumbency and eventually walk (Phase 8) (Figure 1). In this study, 1x MAC of sevoflurane was defined as an end-tidal sevoflurane (Et-sevo) concentration of 2.1%, based on a value from a previous study [20].

Continuous processed EEG data were collected in 2 s epochs, while cardiorespiratory vital signs, including electrocardiography (ECG), HR, respiratory rate, non-invasive systolic, diastolic, and mean arterial blood pressure (NIBP), hemoglobin saturation of oxygen with pulse oximetry (SpO_2_), end-tidal CO_2_ (EtCO_2_), end-tidal sevoflurane (Et-sevo) concentration, body temperature, and subjective anesthetic “depth” scores (Appendix A), were recorded every 3 minutes. In addition, a noxious stimulus via nerve stimulator was also applied after all the cardiovascular data were collected. The raw EEG data were recorded and saved as .EDF files at a sampling rate of either 178 Hz or 89 Hz [21]. Before processing, the sampling rates of all EEG data were adjusted to 89 Hz through appropriate filtering and downsampling [21].

The treatment timeline, detailed below and depicted in Figure 1, consisted of the following phases:

Phase 0: Awake Baseline—Prior to treatment, a short baseline measurement of the dog’s EEG, HR, and NIBP was obtained while the dog was fully awake.

Phase 1: Face Mask Induction—Sevoflurane was administered via a face mask to induce anesthesia. During this phase, the EEG was continuously monitored to observe the changes during the transition from consciousness to unconsciousness. Once endotracheal intubation was completed, the dog was placed on a mechanical ventilator, marking the end of this phase.

Phase 2: Profound Anesthesia—A deep level of anesthesia was initiated by increasing the anesthetic dose until the Et-sevo concentration reached 2.5x MAC (5.3%). To mitigate the profound anesthetic effect, this phase was limited to 10 min of maintenance, contrasting with the remaining phases, which were maintained for 15 min.

Phase 3: Deep Anesthesia—The level of anesthesia was reduced to a deep plane of 2x MAC (4.2% Et-sevo) and maintained at this concentration for 15 min.

Phase 4: Surgical Plane—The level of anesthesia was further reduced to a surgical plane, maintained using a concentration of 1.5x MAC (3.2% Et-sevo). This phase also lasted 15 min.

Phase 5: Light Anesthesia—The level of anesthesia was further reduced to light anesthesia of 1x MAC (2.1% Et-sevo), maintained during this phase for 15 min.

Phase 6: Minimal Anesthesia—The level of anesthesia was reduced to a minimal level of 0.75x MAC (1.6% Et-sevo) and maintained for 15 min.

Phase 7: Early Recovery—Sevoflurane administration was terminated, marking the start of this phase, until the dogs were extubated, which marked the end of phase 7. The EEG, behavioral, and cardiorespiratory parameters were recorded in this phase.

Phase 8: Late Recovery—After the dogs were extubated, they were allowed to recover until regaining walking ability. Recovery behavior was noted for any untoward signs such as thrashing, paddling, and vocalization, with or without salivation. EEG parameters and cardiorespiratory parameters were recorded in this phase. Electrical stimulation was not applied in this phase.

### 2.3. Face Mask Induction, EEG, Cardiorespiratory, Analgesic, and Behavioral Monitoring

#### 2.3.1. Awake Baseline and Sevoflurane Face Mask Induction

Before sevoflurane face mask induction, baseline vital signs, including ECG, heart rate, non-invasive blood pressure, respiratory rate, body temperature, and EEG, were briefly recorded for each dog while awake. Thereafter, the dogs were gently restrained and induced with 8% sevoflurane via face mask using a non-rebreathing circuit (Jackson Reese breathing circuit, Jorgensen Laboratories, LLC., Loveland, CO, USA) with 100% oxygen at a flow rate of 4 L/min. Endotracheal intubation was performed when an appropriate anesthetic level was achieved, as indicated by the absence of toe pinch withdrawal, loss of righting reflex, relaxed jaw and muscle tone, sluggish palpebral reflex, and lack of tongue withdrawal response.

Following endotracheal intubation, the dog was connected to a mechanical ventilator (Hallowell EMC 2002, Pittsfield, MA, USA) using a semi-closed breathing circuit. Ventilator settings, including tidal volume (12–15 mL/kg), respiratory rate (8–13 breaths/minute), and peak airway pressure (13–20 cm H_2_O), were adjusted to maintain an end-tidal CO_2_ between 35 and 45 mmHg. After the dog was stable on the ventilator, EEG and cardiorespiratory data were collected. All dogs received balanced electrolyte intravenous fluids (Plasma-Lyte, Baxter International Inc., Deerfield, IL, USA) at a rate of 5 mL/kg/hour. Additional bolus doses of 5 mL/kg (administered within 5 min) were administered as needed (1–2 times) to alleviate hypotension during the maintenance phase.

#### 2.3.2. Electroencephalography Instrumentation

To record the EEG data, each dog was gently restrained while awake, and six subdermal needle electrodes were positioned on their head according to a modified human 10–20 system, similar to the placement used in our previous canine studies [19]. This was performed prior to anesthetic induction. Specifically, the R1 electrode was placed at Fp2, and the L1 electrode was located at Fp1. The R2 electrode was situated midway between F4 and F8, while the L2 electrode was placed midway between F3 and F7. A ground electrode was positioned on the mid-sagittal central line, and a reference electrode was placed cranially on the midline (Figure 1). Before proceeding, impedance checks were performed on these electrodes using the SedLine^®^ monitor (Masimo Corporation, Irvine, CA, USA) to ensure proper functioning.

#### 2.3.3. Cardiorespiratory Monitoring

To monitor cardiorespiratory data, lead II ECG, SpO_2_ (measured at the tongue or a toe web), and non-invasive blood pressure (systolic, mean, and diastolic) were recorded every three minutes using a cuff sized to 40% of the circumference of one of the hind limbs. These measurements were taken with a multiparameter monitor (Digicare LW9XVet, Lifewindow, Digicare Biomedical Technology, Boynton Beach, FL, USA). Respiratory rate, EtCO_2_, and Et-sevo were continuously monitored via side-stream capnography. Rectal temperature was also recorded and maintained within normal ranges (37.5 °C to 39.2 °C or 99.5 °F to 102.5 °F). Blood pressure, heart rate, and subjective central nervous reflex assessments were used to evaluate the anesthetic level (Appendix A).

#### 2.3.4. Antinociceptive Assessment

To evaluate the antinociceptive effects, two 25-gauge needles were positioned subcutaneously over the lateral aspect of the tibia, five centimeters apart, and connected to a transcutaneous electrical nerve stimulator (SunStim Nerve Stimulator, Ministim, Tri-Animal Health Services, Dublin, OH, USA). A 0.22 ms square-wave pulse stimulus at 400 V with a frequency ranging from 0 to 100 Hz over 3 s was administered, similar to a previous study [19]. The intensity of the stimulus was increased until the animal exhibited a purposeful movement, such as swallowing, head shaking, or purposeful limb movement, or until the maximum stimulation was reached. The antinociceptive assessment was performed every three minutes after the collection of physiological data.

#### 2.3.5. Behavioral Assessment of the Anesthetic Level

A subjective anesthetic “depth” score was assigned to the dogs every 3 min after EEG and cardiovascular parameters were collected. This assessment used a subjective “depth” score system (Appendix A) from our previous study [19]. Auditory responsiveness was also assessed using a clicker, with a positive response defined as the dog turning its head towards the sound source or exhibiting ear and facial muscle twitching. These assessments were performed throughout the study as part of evaluating anesthetic levels. During the subjective depth assessment, jaw tone was evaluated through manual manipulation, while palpebral and corneal reflexes were assessed by applying saline drops (0.9% Sodium Chloride Injection, USP, Baxter International Inc., Deerfield, IL, USA) to the eyelids and cornea to elicit a response. Recovery quality was assessed using a simple descriptive scale, categorizing recovery as smooth, acceptable, or rough. A smooth recovery was defined as uneventful, acceptable recovery required verbal assurance or gentle restraint to ensure calmness, and rough recovery that required significant effort with physical restraint to prevent repeated struggling, paddling, and vocalization.

### 2.4. EEG Data Acquisition, Analysis, and Correlation with Cardiovascular Parameters

The EEG data, including PSI, SR, electromyography (EMG) activity, 95% Spectral Edge Frequency (SEF95), and artifact (ART) activity, were continuously collected during the study and stored as CSV files by the SedLine monitor. The raw EEG data were also obtained for visual examination using EDF files. For EMG activity, the SedLine monitor uses the same forehead electrodes as for EEG to quantify facial muscle activity, incorporated into the PSI calculation, a marker of anesthesia and relaxation.

Statistical analyses included comparing mean values for PSI, SR, SEF95, EMG, and ART across experimental phases using linear mixed models with repeated measures (SAS 9.4, SAS Institute Inc., Cary, NC, USA). Statistical significance was determined at a *p*-value < 0.05, with post hoc comparisons and Bonferroni correction applied as needed. Results are presented as mean ± standard deviation for hemodynamic and EEG parameters in each phase. To explore the association between PSI and cardiovascular parameters (HR and MBP) across varying anesthetic levels, Spearman’s rank correlation coefficients (ρ) were calculated.

## 3. Results

### 3.1. Changes in EEG Indices with Varying Sevoflurane Anesthetic Concentrations (Phases 0–8)

**Figure 2:**
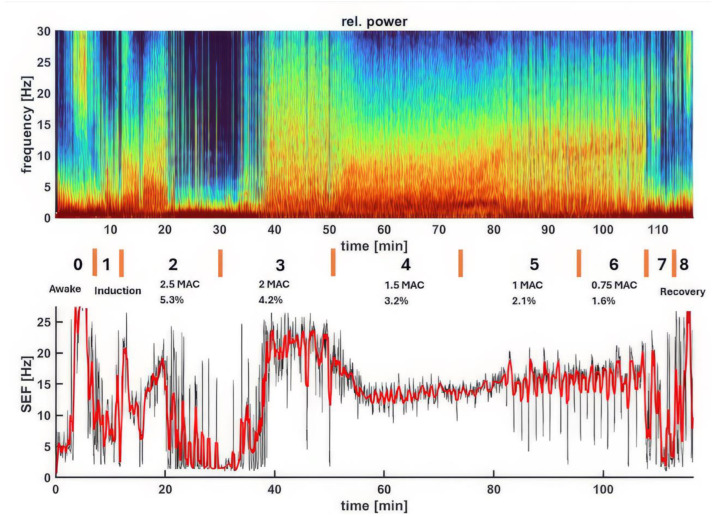
The dynamic changes in brain activity of a representative dog during sevoflurane anesthesia, as shown in the spectrogram (upper panel) and the corresponding SEF95 (lower panel). The spectrogram depicts the power (color scale) and frequency (Hz, *y*-axis) of EEG activity over time (*x*-axis), with color changes reflecting shifts in brainwave frequencies and power. EEG spectrogram power is represented by color: blue (low), teal/green, orange (moderate), and red (high). SEF95 displays median (red) and range (black) values. The phases of anesthesia are indicated by the numbers with descriptions, and orange lines connect the two panels. In the awake state (Phase 0), high-frequency, low-amplitude activity is evident, with SEF95 reaching a peak of 30 Hz. As sevoflurane concentration increases during induction, a shift towards lower frequencies and higher amplitudes occurs. Deep anesthesia (2.5x–2x MAC, Phases 2–3) is characterized by prominent burst suppression (dark blue regions) and delta waves, indicative of significant cortical suppression. Surgical anesthesia (1.5x–1x MAC, Phases 4–5) shows high power in alpha and low beta frequencies that are caused by persistent burst suppression with high alpha and low beta power in the bursts. As sevoflurane concentration decreases, higher frequencies re-emerge, reflecting lighter anesthesia. Following extubation, the EEG gradually returns to the awake pattern. Together, the spectrogram and SEF95 provide a comprehensive view of the brain state changes throughout the anesthetic period.

The progression of EEG indices across the experimental phases is detailed in Table 1. Significant changes in EEG indices were observed with varying sevoflurane anesthetic MAC multiples, showing notable differences between the phases (*p* < 0.001). Figure 2 displays a spectrogram and the associated SEF95 from an example dog, illustrating changes in brain activity under varying depths of sevoflurane anesthesia. As anesthesia deepens (from Phase 0—awake, to Phases 2–6—surgical anesthesia), the dominant power EEG shifts to lower frequencies with larger amplitudes, eventually becoming dominated by burst suppression (the dark blue blocks in the spectrogram) during the profound and deep planes of anesthesia. This then transitions to a typical sevoflurane pattern upon return to the surgical plane. The spectrogram colors and SEF95 changes effectively reflect the dog’s brain state throughout the experiment, from awake to deep anesthesia, back to the surgical plane, and finally to recovery.

**Figure 3:**
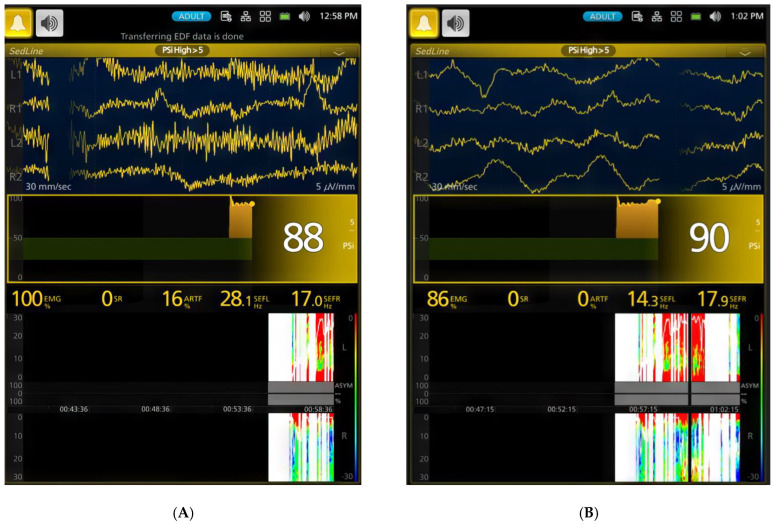
Changes in EEG activity and indices observed in a dog during sevoflurane anesthesia induction. (**A**) (time stamp 12:58 PM). The raw EEG and EEG indices indicate an awake and alert dog. The EEG pattern shows typical low-amplitude, high-frequency activity. The PSI, a processed EEG parameter reflecting the level of consciousness, is consistently high at 88, confirming the dog’s alert state. The SEF95, representing the frequency below which 95% of the total EEG power is located, suggests a relatively high-frequency EEG, with SEF-R at 28.1 Hz and SEF-L at 17.1 Hz. The spectrogram visually represents the power distribution across different frequencies, revealing a predominance of high frequencies (beta and gamma range) characteristic of an awake state. The EMG, at 100%, shows significant activity, reflecting expected muscle tone. The PSI trend graphic, based on the 0–100 scale, uses color to show hypnotic state: dark green zone (25–50) is optimal for general anesthesia, yellow zone indicates near-awake, and blue zone indicates profound anesthesia. The yellow dot shows the heading of the current PSI trend. (**B**) (time stamp 1:02 PM). Halfway through face mask induction with sevoflurane, the raw EEG and EEG indices change, signifying the onset of anesthesia. While the EEG pattern still shows the low-amplitude, high-frequency waveform, there is a drastic reduction in muscle activity and the presence of delta waves. Although the PSI value (90) remains similar, SEF95 begins to decrease, with the SEF-L value dropping to 14.3 Hz. This decrease in SEF95 and EMG activity, along with the changes observed in the spectrogram, indicates the transition from an awake state to an anesthetized state.

**Figure 4:**
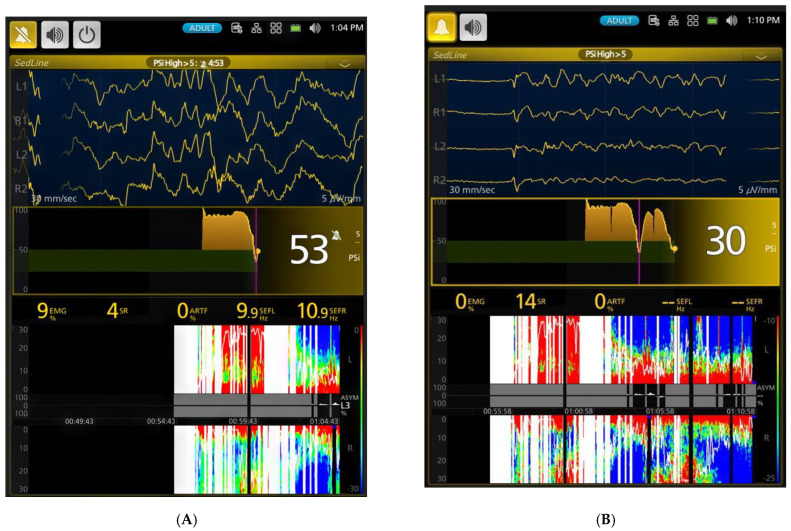
(**A**) (1:04 PM) shows the EEG taken shortly after the dog was intubated. Anesthesia was induced with sevoflurane administered via a face mask. The EEG reveals a mix of delta, theta, and alpha waves, indicating a transition from induction into a surgical plane of anesthesia. The PSI trend graph illustrates a notable decrease from the previous range of 90 (Figure 3B) to 53, which coincides with intubation. This reduction in PSI, alongside SEF95 values (SEF-L at 9.9 Hz and SEF-R at 10.9 Hz), indicates a shift towards lower frequencies with higher amplitude, characteristic of anesthetized brain activity. The spectrogram visually confirms these findings, displaying a significant increase in power within the 0 to 12 Hz range (highlighted in red) and a corresponding decrease in the power of high-frequency waves above 12 Hz (shown in blue). This pattern reflects the deepening of anesthesia, as brainwave activity transitions towards slower, higher-amplitude rhythms. EMG activity is very low at 9%, indicating profound muscle relaxation. (**B**) (time stamp 1:10 PM) captures the dog transitioning to a deeper plane of sevoflurane anesthesia (2.5x MAC-5.3%) with burst suppression in the EEG waves. The EEG displays the classic burst suppression pattern, where periods of high-voltage activity alternate with flat lines. The PSI has now fallen to a value of 30, accompanied by a complete absence of EMG and ART activity. The PSI trend graph effectively illustrates the initial lighter anesthesia immediately after intubation, followed by a gradual deepening as the PSI steadily declines. This transition is mirrored in the spectrogram, where an initial band of higher frequency activity is sandwiched between zones of lower frequencies, reflecting fluctuating anesthetic levels before stabilization.

Anesthesia was induced via a facemask, and endotracheal intubation was successfully performed in all six dogs within an average of 4 min and 18 s (range: 2–5 min). Upon induction, PSI and EMG significantly decreased (Table 1, *p* < 0.001) compared to the awake state (Figure 3A,B), marking the onset of anesthesia (Figure 4A,B).

**Figure 5:**
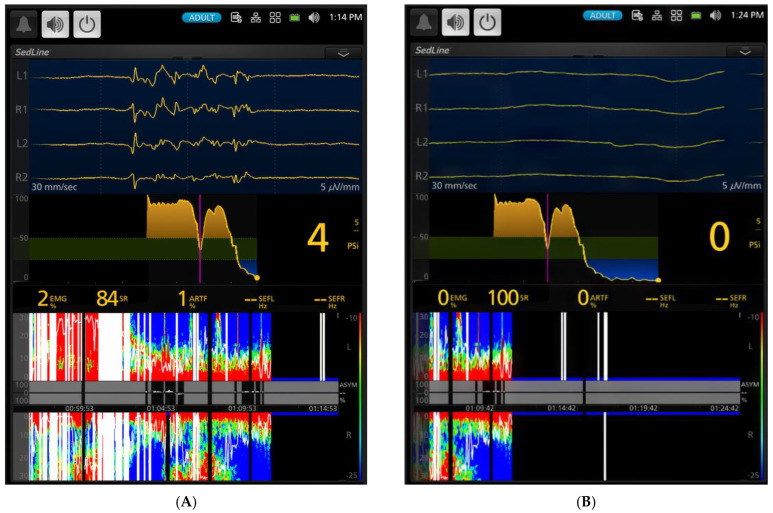
Illustrates a deep plane of anesthesia with profound depression of brain activity in the same study dog exposed to 2.5x MAC (5.3%) of sevoflurane. The EEG waveform transitions from burst suppression ((**A**), time stamp 1:14 PM) to a complete isoelectric pattern ((**B**), time stamp 1:24 PM). Correspondingly, the burst suppression ratio increases from 84% to 100%, and the PSI decreases from a value of 4 to 0. The PSI trend graphic (the middle pannel), based on the 0–100 scale, uses color to show hypnotic state: dark green zone (25–50) is optimal for general anesthesia, yellow zone indicates near-awake, and blue zone indicates profound anesthesia. The yellow dot shows the heading of the current PSI trend. The PSI trend graph further confirms this progression, with PSI values approaching their lowest point.

**Figure 6:**
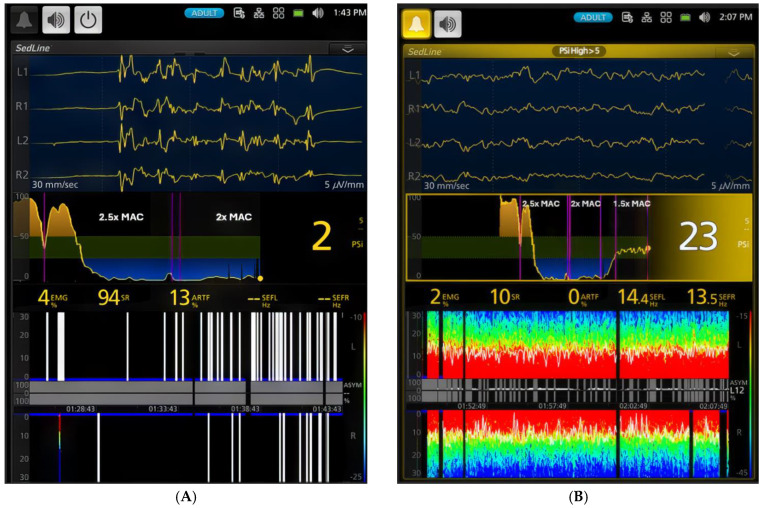
A comparison between a deep plane of anesthesia ((**A**), 2x MAC [4%]) and a surgical plane of anesthesia ((**B**), 1.5x MAC [3%]) in the same dog. The duration of anesthesia at different levels (2.5x, 2x, and 1.5x MAC) is labeled between the vertical pink lines. The small gaps between these lines represent transitions between anesthetic phases. During the deep plane of anesthesia, the dog exhibits a high percentage (94%) of burst suppression in the EEG pattern with a PSI value of 2. The spectrogram demonstrates burst suppression and artifact (13%). When the anesthesia is lightened to a surgical plane, the EEG pattern shifts to a typical sevoflurane anesthesia pattern with slow frequency oscillations dominating the EEG (SEF95—14.4 Hz and 13.5 Hz). The PSI value rises from 2 to 23, and burst suppression decreases from 94% to 10%, with no artifact. The spectrogram now shows vibrant colors, indicating the power of delta, theta, alpha, and low beta frequencies. The PSI trend graph in (**B**) illustrates how PSI values changed over time, from the awake state through induction and sevoflurane maintenance to the surgical plane of anesthesia. This figure clearly demonstrates the ability of PSI to track the level of sevoflurane anesthesia according to its MAC multiple in this dog. The PSI trend graphic (the middle pannel), based on the 0–100 scale, uses color to show hypnotic state: dark green zone (25–50) is optimal for general anesthesia, yellow zone indicates near-awake, and blue zone indicates profound anesthesia. The yellow dot shows the heading of the current PSI trend.

As the anesthesia deepened to the profound level (2.5x MAC) in Phase 2, PSI reached its nadir (13.5 ± 9.9), while SR% peaked (52.7 ± 35.4%). The EEG showed significantly depressed brain wave activity during these phases, evident in the high burst suppression percentages shown in Figure 5A,B and Table 1. This profound suppression allowed the background noise of artifacts to become evident (Table 1). As the anesthetic concentration decreased from profound to surgical plane anesthesia (1.5x MAC), PSI increased, and both SR% and ART% decreased significantly (Figure 6A,B). The EEG patterns at this stage were characterized by dominance of alpha and low beta waves, with an occasional admixture of delta- and theta-band activity. The dogs maintained a high tolerance to electrical stimulation throughout these phases. During profound anesthesia (2.5–2x MAC), a distinct evolution of burst suppression patterns was observed. Initially, a regular pattern with short pauses between bursts emerged. With increasing anesthetic levels, these pauses lengthened while the burst periods shortened, eventually leading to an isoelectric EEG. As sevoflurane concentration decreased and anesthesia lightened to a surgical plane, this pattern reversed, with burst suppression frequency gradually decreasing.

**Figure 7:**
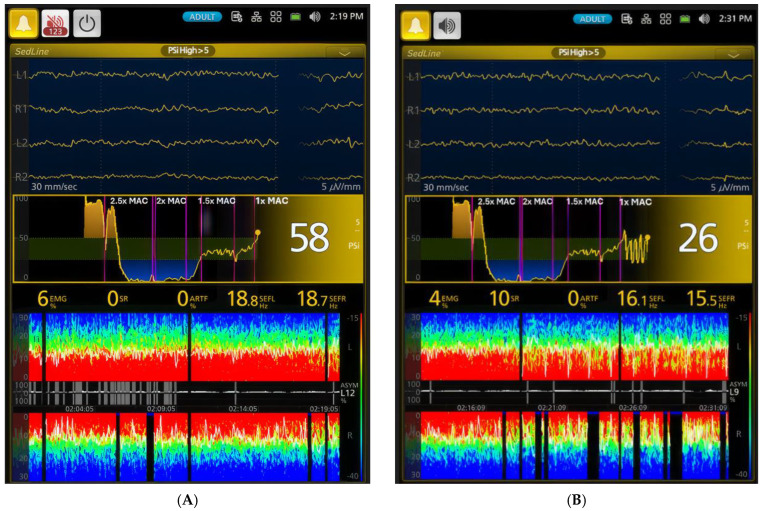
Distinct brain states within the light plane of anesthesia are illustrated. (**A**) depicts a lighter plane of anesthesia than Figure 6B, evidenced by a higher PSI value of 58 compared to 23 in Figure 6B. Furthermore, the SEF95 values are higher, (18.8 and 18.7 Hz), with no burst suppression. While the EEG waveform and spectrogram appear similar to the surgical plane of anesthesia in Figure 6B (1.5x MAC), the PSI trend graph shows an upward shift, indicating a lighter plane of anesthesia with less brain activity depression. In contrast, (**B**) captures an interesting phenomenon: the dog oscillates between lighter and deeper planes of anesthesia while maintained at 1x MAC (2.1%) sevoflurane. During the deeper periods, the PSI decreases to 26, and the burst suppression ratio increases to 10% from 0%. Although the SEF values remain similar, the spectrogram reveals a unique oscillation pattern with distinct red and green colors in both hemispheres and intermittent burst suppression (black blocks with blue tips). This oscillation highlights the dynamic nature of anesthesia even at a seemingly stable MAC. The PSI trend graphic (the middle pannel), based on the 0–100 scale, uses color to show hypnotic state: dark green zone (25–50) is optimal for general anesthesia, yellow zone indicates near-awake, and blue zone indicates profound anesthesia. The yellow dot shows the heading of the current PSI trend.

When anesthesia was reduced to 1x MAC and 0.75x MAC, PSI continued to increase, reflecting a transition from deep to light anesthesia (Table 1). At this lighter plane of anesthesia, the SR% was minimal, and the EEG pattern was dominated by alpha and low beta waves (Figure 7A,B), with a similar admixture of delta and theta waves as observed earlier in the surgical plane of anesthesia.

Once the sevoflurane vaporizer was turned off, the dogs entered Phase 7. One dog was extubated during the transition from 1x MAC to 0.75x MAC. The remaining dogs took an average of 2.8 min (range: 1–5 min) to demonstrate readiness for extubation, characterized by hyperventilation, coughing against the endotracheal tube, and bucking the ventilator. The Et-sevo concentrations just before extubation were 1.9%, 1.5%, 1.6%, 1.5%, 1.5%, and 1.5% for each of the six dogs. Notably, the dog with the highest Et-sevo concentration was the one extubated during the transition to 0.75x MAC.

**Figure 8:**
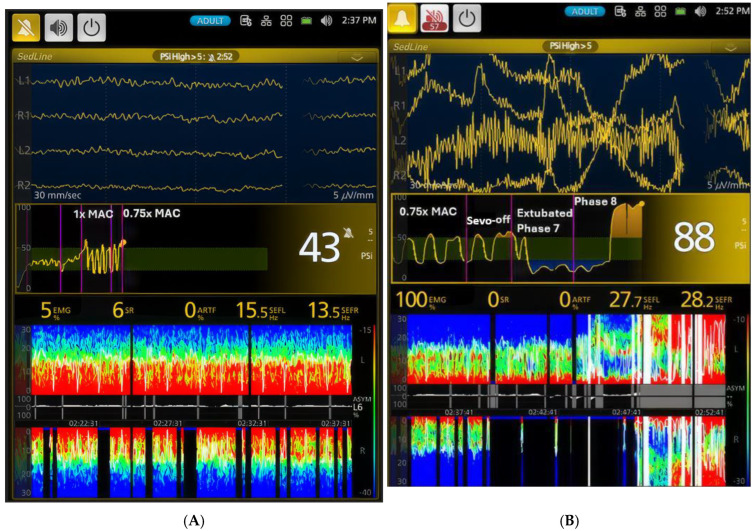
Dynamic changes in brain activity during the transition from anesthesia to recovery are illustrated. (**A**) shows the continued oscillation between deeper and lighter planes of sevoflurane anesthesia as the concentration is reduced from 1x MAC to 0.75x MAC. During the lighter periods, the PSI value increases to 43, and the SEF values remain in the low beta frequency range. The unique oscillation pattern with distinct red and green colors and intermittent burst suppression observed during 1x MAC persists. However, the anesthetic dynamic shifts dramatically when the dog is extubated (Phase 7). The dog enters a sedative state with pronounced burst suppression. As the dog enters Phase 8 (**B**), it transitions from an in-and-out of sedation state to a state with a drastic increase in muscle activity and high beta and gamma brainwave frequencies. The PSI value returns to the awake baseline (88), and the spectrogram displays an awake pattern. These changes are clearly demonstrated in the PSI trend graph, highlighting the rapid shift in brain activity during recovery from anesthesia. The PSI trend graphic (the middle pannel), based on the 0–100 scale, uses color to show hypnotic state: dark green zone (25–50) is optimal for general anesthesia, yellow zone indicates near-awake, and blue zone indicates profound anesthesia. The yellow dot shows the heading of the current PSI trend.

Following extubation, the dogs appeared to revert to a sedative state, as indicated by the reappearance of burst suppression (Figure 7B and Figure 8A) and a decrease in PSI values between Phases 6 and 7 (Table 1). During Phase 7, the dogs remained under the influence of residual sevoflurane (Figure 8A). This effect persisted into the early stages of Phase 8.

Phase 8 marked the duration from extubation to the dogs’ readiness to walk. Recovery quality was assessed using a standardized scale (see the Section 2) based on the level of excitation, vocalization, and required restraint. On average, this took 7.7 min (range: 5–11 min). Initially, the dogs were calm, but as they progressively awakened, their recovery behavior became more pronounced, particularly in the final minutes of Phase 8 (Figure 8B). Four of the six dogs exhibited slight excitation and were rated as having an acceptable degree of recovery, while one dog was rated as having a rough recovery, and one had a smooth recovery. The acceptable recovery was characterized by occasional vocalization, periodic episodes of one–three struggles, and required gentle restraint. The rough recovery dog necessitated longer periods of restraint and was accompanied by more frequent vocalization. Although the vocalization was not loud, it was whiny and of short, discrete duration. Most dogs were walking relatively normally, with slightly wobbly gaits that quickly recovered to normal.

Figure 3, Figure 4, Figure 5, Figure 6, Figure 7 and Figure 8 display screenshots of EEG waveforms, EEG indices, PSI trend graphs, and spectrograms during various anesthetic levels in the same representative dog as shown in Figure 2. These figures clearly demonstrate the transitions in EEG patterns and indices from induction to deep anesthesia, then from deep to light planes of anesthesia, and ultimately to recovery.

### 3.2. Cardiovascular Parameters, Tolerance to Electrical Stimulation, and Subjective Anesthetic Depth Scores of the Dogs During the Various Levels of Sevoflurane Anesthesia

Table 2 presents the Et-sevo anesthetic concentration, cardiovascular parameters, nociceptive assessment, and subjective anesthetic “depth” score for all experimental phases (0–8). Statistically significant differences were observed in oscillometric blood pressure (systolic, diastolic, and mean) across the phases (*p* < 0.001).

In Phase 0 (awake baseline), both HR and MBP were within normal ranges, recorded at 113.6 ± 19.1 bpm and 120.9 ± 24.7 mmHg, respectively. During sevoflurane induction (Phase 1), HR increased to 138.1 ± 13.6 bpm, while MBP decreased significantly to 65.6 ± 19.8 mmHg (*p* < 0.001). Tolerance to electrical stimulation was maximal, and the subjective “depth” score indicated a medium anesthetic plane (3.0 ± 0.0).

As the end-tidal sevoflurane concentration increased in Phase 2 (2.5x MAC), HR remained stable, but MBP continued to decrease, reaching its lowest value at 42.2 ± 7.4 mmHg. Electrical stimulation tolerance remained high, but the subjective “depth” score decreased to 1.8 ± 0.8, reflecting a very deep anesthetic plane.

In the subsequent experimental phases (Phases 3–4), as anesthesia lightened, HR remained similar, but MBP increased significantly (*p* < 0.001), reaching normal values (90.8 ± 25.8 mmHg) in Phase 4. Tolerance to electrical stimulation remained consistent across Phases 2–4. The subjective “depth” score increased as the anesthetic level decreased, reaching 2.9 ± 0.4 in the surgical plane of anesthesia (Phase 4—1.5x MAC).

At light and minimal anesthetic levels (1x MAC—Phase 5 and 0.75x MAC—Phase 6), MBP remained stable (119.9 ± 17.7 mmHg and 119.5 ± 11.1 mmHg, respectively). HR increased slightly in Phase 5 and then decreased to near baseline values in Phase 6. Tolerance to electrical stimulation began to decrease in Phase 5 (772 ± 216.6 Hz), indicating a lower anesthetic level. The subjective “depth” score reflected the decrease in end-tidal sevoflurane concentration, reaching 4 ± 0.3 when the end-tidal sevoflurane concentration was 1.6 ± 0.1% in Phase 6.

When sevoflurane delivery was terminated and the dogs entered the early recovery phase (Phase 7), HR increased to 145.3 ± 26.4 bpm, but MBP remained similar to baseline values (124.8 ± 16.9 mmHg). Tolerance to electrical stimulation continued to decrease, and the “depth” score increased, indicating the dogs’ transition to consciousness. In Phase 8 (late recovery), HR and MBP remained similar to the previous recovery phase. Electrical stimulation was not performed.

Spearman’s rho (ρ) correlation coefficients were calculated to assess the relationships between PSI, HR, and MBP during varying sevoflurane anesthetic levels in each phase. The analysis revealed a weak negative correlation between PSI and HR (ρ = −0.0902) with a *p*-value of 0.5938. In contrast, PSI and MBP demonstrated a moderate positive correlation (ρ = 0.4839) with a *p*-value of 0.0027, suggesting a statistically significant relationship. Additionally, the correlation between HR and MBP was extremely weak (ρ = −0.0089) and not statistically significant, with a *p*-value of 0.9581.

## 4. Discussion

Our study aimed to investigate the EEG indices and patterns associated with different levels of anesthesia induced by sevoflurane in dogs. Awake canines exhibited a high-frequency, low-amplitude EEG pattern with a PSI of 88.8 ± 8.8. As sevoflurane concentration increased during the induction phase, EEG patterns transitioned to lower frequencies and larger amplitudes, with a corresponding decrease in PSI to 56.4 ± 23.3 and the presence of SR. This transition reflects the gradual suppression of cortical activity as the level of anesthesia increases [9,10,11,12,22,23,24,25].

During surgical anesthesia (1.5x–1x MAC), we observed the typical EEG signature with dominating slow frequency activity (i.e., delta, theta, and alpha band activity), with PSI values of 31.8 ± 6.9 and 41.9 ± 17.4, respectively, and SR within 2–7%. This EEG pattern indicates an anesthetic state, where cortical activity is sufficiently suppressed to prevent awareness but not to the extent of inducing a high percentage of burst suppressions [22,23,24,25]. The non-responsiveness to electrical stimulation during this time is evidence of antinociceptive activity. Following extubation, EEG patterns gradually returned to a state similar to the awake EEG, with high frequency and low amplitude and increasing PSI and minimal SR values. These EEG patterns and indices are very similar to those observed in our previous report with isoflurane in dogs [19].

Burst suppression is an EEG pattern characterized by alternating periods of high-voltage activity and electrical silence, a hallmark of profound anesthesia that can progress to an isoelectric EEG, indicating near-complete suppression of cortical activity [26,27,28,29]. This phenomenon is attributed to a marked reduction in cortical neuronal activity, likely mediated by enhanced GABAergic neurotransmission and subsequent reduction in neuronal firing induced by high sevoflurane concentrations [26,27,28,29]. In our study, increasing sevoflurane concentration to induce profound anesthesia (2.5–2x MAC) led to a distinct evolution of burst suppression; initial short pauses between bursts progressively lengthened while burst periods shortened, culminating in an isoelectric EEG. This reflects the progressive impairment of neuronal calcium regulation, with each burst depleting extracellular calcium to levels that inhibit synaptic transmission [26,27,28,29]. During the suppression period, neuronal pumps restore calcium, enabling the next burst. However, as anesthesia deepens, this regulation becomes increasingly impaired, resulting in shorter bursts and longer suppression periods [26,27,28,29]. This process reversed as sevoflurane concentration was lightened.

In the current study, the profound alterations in brain activity were reflected in the EEG indices. During deep anesthesia (2.5–2x MAC), PSI values reached their nadir at 13.5 ± 9.9 and 18.6 ± 15.8, while SR values peaked, reflecting the near-silence of the EEG. This observation aligns with a previous canine study where burst suppression was reported at a similar sevoflurane concentration at 2.14x MAC [20]. In our study, burst suppression occurred across various MAC multiples, but was significantly higher during 2.5x and 2x MAC (Table 1), peaking at 52.7 ± 35.4%, with some dogs exhibiting 100% burst suppression.

In this study, sevoflurane effectively induced unconsciousness and unresponsiveness in the dogs at surgical MAC levels and beyond, as evidenced by the profound suppression of neuronal activity observed in the EEG. This suppression disrupts the brain’s integrative functions, leading to a state where the animals lack awareness and responsiveness to external stimuli, including those that would normally be perceived as noxious. These findings align with our previous study using isoflurane at comparable MAC multiples [19], where similar patterns of unconsciousness were observed. This observed suppression of neuronal activity likely arises from sevoflurane’s complex interactions with various neuronal receptors and ion channels. The key receptors and neuronal activities involved include the gamma-aminobutyric acid (GABA) receptors, N-Methyl-D-Aspartate (NMDA) receptors, and others [22,23,24,25]. By modulating neuronal excitability and neurotransmission, these interactions contribute to the hypnotic effects of sevoflurane [22,23,24,25]. Further research is needed to fully elucidate the intricate mechanisms by which sevoflurane produces these profound effects on brain activity and consciousness.

Comparing our findings to previous studies in humans [30] and dogs [31] reveals similarities and differences in the hemodynamic response to sevoflurane. Similarly to the human study [6,30,32,33], we observed a decrease in blood pressure with increasing sevoflurane concentrations, while the heart rate remained relatively stable. This consistency across species underscores the vasodilatory effects of sevoflurane, which reduces systemic vascular resistance and consequently lowers blood pressure [6,30,31,32,33].

Interestingly, some key differences emerged in how heart rate responded to sevoflurane. While human studies show stable heart rates under sevoflurane anesthesia, a previous canine study [31] found that heart rate actually increased at 1.2x and 2.0x MAC. In contrast, the current study found no significant heart rate changes between the awake baseline and 2.5x, 2x, and 1.5x MAC.

This difference in response could be due to several factors, including variations in how different species regulate their autonomic nervous system. It is also possible that the dogs in our study, who were not sedated with any premedication, may have experienced some level of stress associated with the experimental setting. This could have led to an elevated baseline heart rate, potentially masking any further increases due to sevoflurane. Additionally, differences in study protocols, such as the use of premedication or the specific anesthetic delivery techniques, might also contribute to the observed variations in heart rate response.

Previous research in dogs [31] has shown that while heart rate might increase under sevoflurane anesthesia, cardiac output can significantly decrease at higher MAC levels, particularly at 2.0x MAC. This finding is particularly relevant to our study, where heart rate remained stable across increasing MAC levels. It suggests that relying solely on heart rate to assess cardiovascular stability under sevoflurane anesthesia can be misleading, as cardiac output might still be compromised even with a stable heart rate. While our study did not directly measure cardiac output, this previous work highlights a potential risk of exceeding 2x MAC, even when the heart rate appears stable.

Interestingly, human studies [6,32,33] have revealed a unique compensatory mechanism involving cardiac index. In these studies, cardiac index initially decreased at lower MAC multiples but returned to baseline at 2.0x MAC in subjects who maintained adequate blood pressure. This suggests that decreased systemic vascular resistance at higher sevoflurane concentrations can help maintain cardiac output despite potential myocardial depression [6,30,31,32,33]. This compensatory mechanism may explain why some human studies show stable hemodynamics under sevoflurane anesthesia, even at higher MAC levels. However, further research is needed to determine whether similar compensatory mechanisms exist in dogs.

The potential for cardiovascular complications at higher MAC levels necessitates vigilant monitoring of both cardiovascular function and brain state status. Excessive anesthetic depth can lead to several adverse consequences, including hemodynamic instability (hypotension, bradycardia, and decreased cardiac output), respiratory depression, delayed emergence from anesthesia, and potentially, postoperative cognitive dysfunction (well documented in human patients). In extreme cases, particularly in critically ill patients, it may even increase the risk of mortality.

In the present study, the young, healthy dogs fortunately withstood the brief hemodynamic challenges (mainly hypotension) associated with the excessive anesthetic depth. This tolerance is likely due to their robust cardiovascular health. Additionally, any respiratory depression was effectively managed with mechanical ventilation. However, it is important to note that only one dog exhibited a smooth recovery, while the others showed varying degrees of delirium and rough recovery. This contrasts with our previous isoflurane study, where five out of six dogs experienced a rough and delirious recovery, potentially linked to excessive anesthetic depth [19]. This difference highlights the possible influence of anesthetic agents on recovery quality, an area that warrants further investigation. These findings underscore the importance of preventing anesthetic overdoses, especially since this study employed anesthetic levels of 2 to 2.5 MAC. The consequences of excessive anesthetic depth could be far more pronounced in diseased or aged animals with compromised physiological reserves.

In this study, we observed that, in addition to blood pressure monitoring, maintaining burst suppression below 10% proved to be an effective strategy for ensuring sufficient anesthesia while promoting heart rate and some blood pressure measurements within normal ranges. This cutoff was based on our observations at 1.5x MAC, where burst suppression ratios remained low, while the MBP and PSI remained within acceptable ranges. These findings suggest that using EEG indices like PSI and burst suppression, in conjunction with blood pressure monitoring, allows for real-time assessment of anesthetic depth and can help prevent excessively deep anesthesia with potential cardiovascular consequences.

We also examined the correlations between MBP, HR, and PSI to further elucidate the relationship between brain activity and cardiovascular parameters during sevoflurane anesthesia. Our analysis revealed a weak negative correlation between PSI and HR (ρ = −0.0902, *p* = 0.5938). In contrast, PSI and MBP demonstrated a moderate positive correlation (ρ = 0.4839, *p* = 0.0027), suggesting that MBP is a more reliable indicator of the level of sevoflurane anesthesia than HR in dogs. This finding aligns with our previous observations with isoflurane [19].

The current study has several limitations. Firstly, using male beagle dogs may limit the generalizability of our findings to other breeds and sexes. Secondly, while our non-invasive blood pressure monitoring provided valuable data, additional invasive monitoring could have yielded more comprehensive hemodynamic information, such as cardiac output and systemic vascular resistance. Finally, our sample size, while adequate for detecting significant changes, may have limited our ability to identify subtle correlations or individual variations in response to sevoflurane. It is also important to acknowledge that this study exclusively used sevoflurane. However, in clinical practice, sevoflurane is often used in combination with other anesthetic agents, such as dexmedetomidine, propofol, and ketamine. These drug interactions could potentially influence the MAC and PSI values, adding another layer of complexity to the interpretation of our results. Further research exploring the combined effects of sevoflurane with other commonly used anesthetic drugs is warranted to enhance the clinical applicability of these findings.

## 5. Conclusions

In conclusion, in this study, we investigated the effects of varying sevoflurane concentrations on frontal EEG patterns in dogs. Our findings demonstrate that frontal EEG can effectively track brain state changes during different anesthetic levels, with distinct patterns emerging at each sevoflurane MAC multiple. We observed clear progression from high-frequency, low-amplitude activity in awake dogs to burst suppression and isoelectricity during profound anesthesia, confirming the utility of frontal EEG in monitoring the anesthetic level. Furthermore, our analysis revealed a moderate correlation between the mean blood pressure and PSI, but not between the heart rate and PSI, suggesting that blood pressure is likely a more reliable indicator of sevoflurane anesthetic levels in dogs.

In addition, this study provides a detailed characterization of EEG patterns across a range of sevoflurane MAC multiples, including quantitative analysis of burst suppression and its evolution with deepening anesthesia. These results contribute to a more comprehensive understanding of sevoflurane-induced anesthesia in dogs, laying a foundation for improved anesthetic protocols and monitoring techniques to enhance patient safety and optimize anesthetic management in real-time.

## Figures and Tables

**Table 1:** The EEG indices changed in association with varying sevoflurane concentrations in the six dogs. The indices changed as the dogs transitioned from an awake state (Phase 0) to face mask induction (Phase 1), through various levels of sevoflurane maintenance anesthesia (Phases 2–6), termination of sevoflurane to extubation (Phase 7), and from extubation to recovery to walking (Phase 8). Statistical analysis showed significant differences (*p* < 0.001) in all EEG indices across the treatment phases. Data are presented as mean ± standard deviation. Different letters within a column denote significant differences, while identical letters signify no significant difference.

Phases	PSI	SR%	SEF-L	SEF-R	EMG	ART
Baseline (0)	88.8 ± 8.8 ^a^	1.9 ± 3.4 ^a^	18.3 ± 9.5 ^a^	12.7 ± 8.8 ^a^	53.5 ± 37.1 ^a^	11.5 ± 17.5 ^a^
Induction (1)	56.4 ± 23.3 ^b^	1.9 ± 3.9 ^a^	9.3 ± 6.1 ^b^	10.7 ± 6.3 ^a,b^	23.4 ± 26.2 ^b^	17.9 ± 22.8 ^b^
2.5x MAC (2)	13.5 ± 9.9 ^c^	52.7 ± 35.4 ^b^	2.7 ± 2.9 ^c^	2.7 ± 3.0 ^c^	6.5 ± 6.5 ^c^	51.2 ± 42.9 ^c^
2x MAC (3)	18.6 ± 15.8 ^d^	35.8 ± 32.9 ^c^	9.7 ± 5.4 ^b^	9.9 ± 5.7 ^b^	6.2 ± 4.7 ^c^	34.9 ± 38.1 ^d^
1.5x MAC (4)	31.8 ± 6.9 ^e^	2.4 ± 9.4 ^a^	10.7 ± 4.3 ^d^	10.1 ± 4.0 ^b^	4.1 ± 3.0 ^d^	0.3 ± 0.9 ^e^
1x MAC (5)	41.9 ± 17.4 ^f^	7.1 ± 13.0 ^d^	11.9 ± 5.5 ^e^	12.3 ± 5.0 ^a^	7.8 ± 14.4 ^c^	0.6 ± 2.7 ^e^
0.75x MAC (6)	46.9 ± 18.2 ^g^	6.5 ± 12.9 ^d^	14.6 ± 5.1 ^f^	12.3 ± 4.6 ^a^	11.2 ± 9.4 ^e^	0.4 ± 2.1 ^e^
Early recovery (7)	32.8 ± 16.8 ^e^	13.1 ± 15.0 ^e^	9.9 ± 5.9 ^b,d^	8.5 ± 5.2 ^e^	23.6 ± 24.5 ^b^	4.7 ± 9.6 ^e^
Late recovery (8)	39.8 ± 27.1 ^f^	13.2 ± 16.7 ^e^	5.9 ± 6.2 ^g^	5.4 ± 5.3 ^f^	44.1 ± 30.6 ^f^	18.9 ± 23.0 ^f^

**Table 2:** The cardiovascular parameters, end-tidal sevoflurane concentration, maximum tolerance to electrical stimulation (Hz), and subjective anesthetic “depth” scores for six dogs during sevoflurane anesthesia are listed. The data span from awake baseline (Phase 0) to face mask induction (Phase 1), through various anesthetic levels indicated by MAC multiples, to extubation (Phase 7) and walking (Phase 8). Data are presented as mean ± SD. SBP, DBP, and MBP denote systolic blood pressure, diastolic blood pressure, and mean arterial blood pressure, respectively. An asterisk (*) indicates a significant difference (*p* < 0.001) between treatment phases. Different letters indicate significant differences within each column, while the same letter signifies no significant difference.

Phase	ET-Sevoflurane Concentration (%)	HR (bpm)	SBP * (mmHg)	MBP * (mmHg)	DBP * (mmHg)	Tolerance to Electrical Stimulation (Hz, Maxim 900 Hz)	“Depth“ Score (1-Deep, 5-Light)
Baseline (0)	N/A	113.6 ± 19.1	162.9 ± 19.9 ^a^	120.9 ± 24.7 ^a^	110.4 ± 28.9 ^a^	N/A	N/A
Induction (1)	5.4 ± 2.5	138.1 ± 13.6	110.3 ± 28.4 ^b^	65.6 ± 19.8 ^b^	60.7 ± 43.2 ^b,d^	900.0 ± 0.0	3.0 ± 0.0
2.5x MAC (2)	5.3 ± 0.2	120.2 ± 12.3	68.4 ± 13.9 ^b,c^	42.2 ± 7.4 ^b,c^	32.7 ± 8.2 ^b,c^	886.9 ± 45.8	1.8 ± 0.8 ^a^
2x MAC (3)	4.2 ± 0.1	120.4 ± 8.5	82.9 ± 22 ^b^	55.5 ± 16.8 ^b^	43.6 ± 13.1 ^b,d^	900.0 ± 0.0	1.9 ± 0.9 ^a,b^
1.5x MAC (4)	3.2 ± 0.1	127.1 ± 13	116.6 ± 16.6 ^b^	90.8 ± 25.8 ^b^	68.8 ± 21.3 ^b^	875.0 ± 76.9	2.9 ± 0.4 ^a,b^
1x MAC (5)	2.1 ± 0.1	134.2 ± 21.8 ^a^	153.4 ± 17.9 ^b,d^	119.9 ± 17.7 ^b,d^	100.9 ± 18.1 ^b,d^	772.0 ± 216.6	3.5 ± 0.5 ^a^
0.75x MAC (6)	1.6 ± 0.1	117.8 ± 17.8 ^b^	155.1 ± 11.1 ^b,d^	119.5 ± 11.1 ^b^	105.7 ± 9.2 ^b,d^	690.0 ± 265.5	4.0 ± 0.3 ^a,b^
Recovery (7)	1.2 ± 0.5	145.3 ± 26.4 ^a,c^	156.8 ± 16.4 ^b,d^	124.8 ± 16.9 ^a,d^	107.4 ± 14.9 ^a,d^	650.0 ± 212.1	4.6 ± 0.5 ^b^
Late recovery (8)	N/A	151.7 ± 24.3 ^a,d^	154.2 ± 12.2 ^b,d^	124.4 ± 15.9 ^a,d^	105.8 ± 16.2 ^a,d^	N/A	5.0 ± 0.0 ^b,c^

## Data Availability

The data are contained within the article.

## Appendix A

**Table A1:** Subjective anesthetic depth scores in dogs. The palpebral reflex was assessed with a cotton swab, and the corneal reflex was assessed by applying sterile saline drops to the corneal surface via a syringe [34].

Anesthetic Plane	Depth Score	Pupil Size	Globe Position	Eye Reflex (P: Palpebral C: Corneal)	Heart Rate, Blood Pressure, Breathing	Third Eyelid Position	Jaw Tone, Body/Limb Movement, or Swallowing
Very light	5	Large or small, moving	Central	P: active C: active	Increased, spontaneous, breathing attempts	Retracted	Strong
Medium-light	4	Large or small	Centra-l or ventromedial	P: mildly depressed C: mildly depressed	Increased, spontaneous breathing attempts	Retracted or partially prolapsed	Medium
Medium	3	Small or medium	Ventromedial	P: markedly depressed C: mildly depressed	Normal	Completely prolapsed	None or barely
Medium-deep	2	Medium	Central	P: absent C: markedly depressed	Normal or depressed	Completely or partially prolapsed	None
Very deep	1	Large	Central	P: absent C: absent	Depressed	Partially depressed	None
